# Does intellectual property protection promote green innovation in firms? A perspective on R&D spillovers and financing constraints

**DOI:** 10.1371/journal.pone.0288315

**Published:** 2023-11-08

**Authors:** Xiaoyuan Gao, Yixin Zhao

**Affiliations:** Law School of Tianjin University, Tianjin, China; East China Normal University, CHINA

## Abstract

Although the literature has assessed the impact of Intellectual property protection on urban innovation, there is still a gap in the assessment of the impact of green innovation at the firm level. This study constructs a multi-period differences-in-differences (DID) model using China’s Intellectual Property Demonstration Cities (IPDC) as a quasi-natural experiment to investigate the impact of IPDC on corporate green innovation. The findings indicate that (1) the IPDC program significantly stimulates corporate green innovation and has long-term effects. This finding still holds after using PSM-DID as well as robust IW estimators. (2) Mechanism analysis suggests that IPDC can promote firms’ green innovation by reducing R&D spillover losses and alleviating financing constraints. (3) Heterogeneity tests show that the IPDC program has a more significant promotion effect on small, state-owned, growth-stage firms. Based on the above findings, this study provides policy implications for enhancing intellectual property protection to stimulate corporate green innovation.

## 1. Introduction

Green innovation plays a vital role in both sustainable economic development and environmental protection [1, 2]. On the one hand, green innovation can improve the production efficiency and competitiveness of enterprises and promote sustainable economic development [3]. On the other hand, green innovation can reduce environmental pollution and resource waste through the development and application of environmentally friendly technologies and products, thus achieving environmental sustainability [4]. As an important economic activity and source of innovation, enterprises play a vital role in promoting green innovation [1]. Based on the carbon peaking and carbon neutrality goals, China’s 14th Five-Year Plan proposes to encourage green innovation and promote the green transformation of enterprises. In April 2019, the National Development and Reform Commission issued the "Guidance on Building a Market-oriented Green Science and Technology Innovation System". Since then, green innovation has become a key task in the current national ecological civilization construction.

However, the effectiveness of patent protection in China is insufficient, which hinders the development of green innovation. As of 2019, only 18.6 percent of China’s patents had survived for 20 years, and the average patent life was 7.6 years, much lower than Thailand’s 14.8 years and the United States’ 9.7 years [5]. Green innovation generally requires significant human and financial resources for a long period of research and development. When property rights are not adequately protected, companies often choose to free-ride on the innovation outcomes of other companies rather than take the initiative to innovate. China Rule of Law Development Report No.15(2017), based on the average investment of more than 7,000 Chinese enterprises in R&D programs, found that only 6.2% of enterprises were willing to conduct long-term research and development for more than three years.

In order to enhance the protection of intellectual property rights, the State Intellectual Property Office launched the Intellectual Property Pilot Demonstration Cities (IPDC) program in 2011 and announced the first batch of selected demonstration cities in 2012. The State Intellectual Property Office provides IPDC with guidance on intellectual property protection and conducts regular assessments of IPDC. On this basis, some literature has evaluated the effectiveness of implementing IPDC programs. Most of the literature focuses on the impact assessment of the IPDC program on urban innovation and development [6, 7]. In addition, a portion of the literature assesses the impact of the IPDC program on firm innovativeness [8, 9]. However, there is still a gap as to whether IPDC has stimulated technological innovation in green aspects of enterprises. To this end, this paper investigates the impact of the IPDC program on firms’ green innovation based on the perspectives of financing constraints and R&D spillovers. In addition, we explore the heterogeneous effects of firm characteristics on IPDC programs.

The marginal contributions of this paper are mainly in the following aspects. First, the impact of the IPDC program on green innovation is assessed at the firm level. To the best of our knowledge, there is still a gap in research on the impact of the IPDC program on corporate green innovation. Our impact assessment of the IPDC program provides a new entry point for the green transformation of enterprises. Secondly, based on the perspective of enterprises’ financing constraints and R&D spillover, the transmission mechanism of the impact of IPDC establishment on enterprises’ green innovation is elucidated to enrich its intrinsic logical connection. Third, the different impacts of heterogeneous firm characteristics on the IPDC program are examined. These results can provide targeted suggestions for the government to promote IPDC protection.

The rest of the paper is organized as follows. The second section is a review of the relevant literature. The third section presents the theoretical analysis and hypotheses of the digital transformation of firms on environmental performance. The fourth section presents the research design, variable selection, and data sources. The fifth section presents the empirical results. The sixth section summarizes the study findings and then makes policy recommendations.

## 2. Literature review

### 2.1 Intellectual property protection

Because of the uncertainty in the invention, production, and application, technological knowledge is often viewed as a potential public good [10]. When producers of technological knowledge make a factor input but users use it without considerable cost, everyone tends to wait to enjoy the benefits of knowledge spillovers. This will eventually lead to a slackening of the production of technological knowledge by all, which is known as free-riding behavior in economics [11]. This phenomenon is particularly evident in firms. Firms inherently exist to pursue profits [12]. Firms will lose the incentive to produce technological knowledge, also known as innovation if they need to invest heavily to produce it while competitors can obtain similar technology without spending the same cost [13]. Therefore, defining and protecting property rights for technological knowledge is essential to stimulate innovation in firms.

The IPDC program in China was established to promote intellectual property protection, improve the use and management of intellectual property rights, and enhance intellectual property publicity and education in the city. The construction of IPDC can promote the protection and application of intellectual property rights and provide guarantee and support for enterprises’ innovation [8]. At the same time, the establishment of IPDC can also enhance enterprises’ awareness and attention to intellectual property rights, and promote enterprises to strengthen the management and application of intellectual property rights [14]. Further, studies have found that the establishment of IPDCs contributes to the economic development of cities [15].

### 2.2 Research on green innovation

Green innovation refers to an innovation that achieves the production and use of products or services by adopting clean technology, thus reducing the pollution of the environment and the consumption of resources [16, 17]. Green innovation encompasses new technologies, products, services, or business models that have a positive impact on the environment and society [18]. Compared to traditional innovation, they have many similarities but also differences [19, 20]. In fact, more than just the pursuit of financial performance, corporate green innovation includes additional goals of improving the company’s sustainability performance and helping the green transformation of the country and society [21]. Green innovation is therefore crucial for developing countries under pressure to transform their economies.

In this regard, the literature has conducted research based on how to promote green innovation. At the macro level, the literature has examined the impact on provincial and prefecture-level green innovation in terms of environmental regulation [22, 23], green finance [24, 25], foreign direct investment [26], and so on. There is also literature that examines the impact of financial performance [27], management characteristics [28], stakeholders [29, 30] and digital transformation [31, 32] on green innovation at the firm level from a micro perspective. In addition, the literature also investigated the impact of China’s policy pilots on firms’ green innovation. For example, Chen, Zhang [33] found that China’s carbon trading rights pilot policy significantly reduced the proportion of green patents. Hu, Wang [34] found that the Green Credit Guidelines (2012 Guidelines) policy issued by China stimulated green innovation among heavy polluters. Wang, Song [35] demonstrated that the low-carbon city pilot encourages companies to improve production technology and enhance green innovation in listed companies.

### 2.3 The impact of intellectual property protection on innovation

The role of enhanced protection of intellectual property in stimulating innovation has been at the center of the debate [36, 37]. The literature generally acknowledges the need to protect intellectual property in order to stimulate firms’ willingness to innovate and sustain long-term economic growth [8, 38]. Sweet and Maggio [39] identified a stronger strong relationship between intellectual property and innovation activity using cross-country panel data. However, some researchers have pointed out that the overprotection of intellectual property can have a negative impact on innovation [40, 41]. In addition, some literature has suggested a non-linear relationship between the strength of intellectual property protection and innovation. For example, Gangopadhyay and Mondal [42] found that intellectual property protection and innovation have an inverted U-shaped relationship, as too much protection limits future innovation.

Research on the innovation effects of the IPDC program in China has focused on the study of urban innovation capacity [6, 7]. Part of the literature examines the green impact of the IPDC program on cities. Mao and Failler [43] showed that the IPDC program significantly increases green total factor productivity in cities. Qin and Gao [44] concluded that the IPDC program promoted the optimization and upgrading of the city’s industrial structure. In addition, some literature has examined the impact of IPDC on firm-level innovation. Zhu and Sun [45] found that the IPDC program promotes technological innovation and thus increases total factor productivity in firms. Fang, Lerner [8] found that the IPDC program promoted innovation and was particularly effective for private companies. Qian, Sun [46] found that the IPDC program would stimulate technological innovation by reducing the risk of intellectual property infringement, increasing the innovation dynamics of the model cities and lowering the barriers to intellectual property pledges. Lv, Pan [47] argued that the IPDC program has reduced the cost and risk of innovation for companies, which in turn has promoted technological innovation.

## 3. Hypothesis development

### 3.1 The mechanism of intellectual property protection on green innovation

#### 3.1.1 R&D spillover loss reduction mechanism

In the process of green innovation, firms may face high R&D costs in developing and applying environmentally friendly technologies and green products. If these technologies and products are copied by other companies or individuals, the original firms’ R&D investments will not be returned, which may discourage firms from innovating in the environmental field. This is often studied in the literature as "R&D spillover", a phenomenon in which the R&D results of one firm are used by other firms or individuals without adequate compensation [48]. R&D spillover is actually an externality problem of innovation activities, i.e., it is difficult for firms to prevent other firms from imitating their intellectual property [49]. When there are serious R&D spillovers in an industry, if the government strengthens intellectual property enforcement protection, it will make firms implementing green innovation in the industry more likely to benefit from patent licensing or patent monopoly use and less likely to harm their interests due to technology infringement. the IPDC program aims to strengthen the protection of intellectual property rights and thus increase firms’ willingness to innovate by reducing R&D spillover losses. Based on the above analysis, we propose:

*Hypothesis 1*: IPDC program stimulates corporate green innovation by reducing R&D spillover losses.

#### 3.1.2 External financing constraint relief mechanism

Successful innovation activities of enterprises depend on adequate external equity and debt financing [50]. Enterprises usually need large amounts of capital to support green innovation, but due to its partly non-profit purpose and high-risk nature [51], many traditional financial institutions are unwilling to provide adequate financing support, resulting in more serious financing constraints for enterprises [52]. The IPDC program would alleviate the pressure on external financing for corporate green innovation in the following two ways.

On the one hand, one of the focuses of the IPDC program is to facilitate intellectual property pledge financing services so that companies can more easily obtain financing support [47]. Enterprises can use their intellectual property as collateral to obtain financing, which can reduce the cost and risk of financing for enterprises and improve their financing ability [45]. IPDC program can also provide intellectual property valuation and trading services, enabling companies to better leverage their intellectual property. Through appraisal and trading, companies can liquidate their intellectual property, which can help them raise more capital for green innovation.

On the other hand, the IPDC program increases the value and protection of a company’s intellectual property by promoting the creation, use, and protection of intellectual property. A firm can gain a higher market share and profit if its environmental technologies and products are protected by law and other firms are not free to use or copy their own technologies and products [53]. This will help attract more investment and capital stock, improve the financing capacity and capital strength of the enterprise, and provide better support and protection for the enterprise’s green innovation. Accordingly, we suggest:

*Hypothesis 2*: The IPDC program stimulates corporate green innovation by relieving financing constraints.

### 3.2 Heterogeneous impact

The magnitude of the effect of IPDC establishment on firm green innovation may vary depending on firm characteristics. The first is the scale of the enterprise. Large-scale enterprises usually have more abundant resources such as R&D funds, technical talents, and market channels. This makes it easier for them to obtain support and recognition from the government and the market, and thus more conducive to green innovation [31]. The intellectual property services and protection provided by IPDC will further enhance the innovation efficiency and results of large enterprises, making it easier for them to gain competitive advantages and economic benefits in the market. In contrast, small-scale enterprises usually have limited resources and relatively weak technical strength, and their investment and practice in green innovation are also lower [54]. Although IPDC can also provide a better innovation environment and support for small firms, it may not be as effective as large firms in promoting green innovation.

Firm ownership has also been identified in the literature as a key factor contributing to differences in innovation decisions [55, 56]. First, privately owned firms are more focused on innovation and intellectual property protection because they typically rely more on innovation to maintain a competitive advantage [57]. In contrast, state-owned ownership firms are typically more dependent on government resources and policy support [58]. In an IPDC program, privately owned firms may be more actively engaged in innovation activities to better protect and apply intellectual properties. Second, privately owned firms are usually more flexible and agile, and can adapt more quickly to market needs and changes [59]. The IPDC program provide a better environment for firms to protect and apply intellectual properties, making it easier for firms to innovate and turn their innovations into marketable products. Privately owned firms may be able to turn innovations into marketable products more quickly than state-owned firms [60], and therefore may have a more competitive advantage in the IPDC program.

Research has been conducted to conclude that there are significant differences in the innovation intentions of firms at different life cycle stages [61]. Therefore, the establishment of IPDC may have heterogeneous effects on the green innovation of firms in different life cycle stages. First, companies in the growth stage pay more attention to innovation and development [62]. IPDC program invests resources to create an innovative ecological environment at the early stage of establishment, such as establishing innovation and business incubators, technology exchange platforms, and innovation funds. The establishment of these innovative ecological environments helps stimulate the green innovation vitality of companies in the growth stage. Second, growth-stage companies have greater financing needs. Although growth-stage companies are willing to innovate, their main task is to gain a firm foothold in the industry [63]. These companies tend to invest their limited capital in the purchase of necessary fixed assets to ensure the normal operation of their main business. Conversely, companies in the mature stage have relatively stable profits and cash flow and are more likely to obtain large amounts of external financing at lower costs [64]. Companies in the decline stage tend to spend more resources on survival than on innovative R&D [65]. Therefore, the establishment of IPDC may better facilitate growth-stage companies with a high willingness to innovate but high financing constraints. Based on the above analysis, we propose:

*Hypothesis 3*: *The IPDC program is a stronger catalyst for green innovation for large-scale*, *privately owned*, *and growth-stage companies*.

## 4. Methodology

### 4.1 Model construction and variables selection

As a pilot policy, the IPDC program was implemented in different cities in 2012, 2013, 2015, 2016, and 2018, respectively. Due to the randomness of the selection of pilot cities, this paper treats IPDC as an exogenous policy shock and builds a multi-period DID model to examine the impact on corporate green innovation. The model is shown as follows:

$${GI}_{ijt}=\alpha +\beta {IPDC}_{j,t}+\gamma X_{jt}+\mu_{i}+\eta_{t}+\varepsilon_{ijt}$$ (1)

where *GI*_*i*,*t*_ denotes the green innovation levels in the firm *i* in city *j* and year *t*. According to the common practice of existing literature [28, 32], the green innovation ability of enterprises is measured by the number of green innovation applications. In this paper, green patent application data of listed companies are used as the explanatory variables. The reasons are as follows: First, green patents most intuitively reflect the output of enterprises’ green technology innovation activities, with quantifiability and spillover from inside and outside the industry. Moreover, compared with R&D inputs, patents have clear technology classification, and the patent data can be further classified according to different technology nature accordingly to reflect the different value connotation and contribution of innovation activities. Second, considering that the patent application process is time-consuming, using patent application data instead of patent grant data can examine the impact of the pilot policy on enterprises’ green technology innovation activities in a more time-sensitive manner. The number of green innovation applications is the sum of the number of green invention patent applications and the number of green utility model patent applications. *IPDC* is a dummy variable that assigns a value of 1 if city *j* is a pilot city in year *t* and 0 otherwise. *μ*_*i*_ and *η*_*t*_ represents the firm fixed effects and year fixed effects. *ε*_*ijt*_ is the random error term. We also control for additional factors that may confound the DID analysis, denoted by *X*_*it*_. With reference to the existing literature [28, 34], we control for firm size (*lnsale*), firm age (*lnage*), whether ownership is state-owned (*property*), leverage (*lev*), cash ratio (*cash*), the shareholding ratio of the first largest shareholder (*herf*), the difference between the shareholding ratio of the first and second largest shareholder (*er*), whether audit work is performed by a Chinese Big Four audit firm (*audit*), total net asset margin (*roa*), board size (*director*), and the number of independent board incumbents (*indepen*).

In addition, to demonstrate that IPDCs have a higher level of intellectual property protection than non-demonstration cities, drawing on Long, Yi [66], we manually compiled the number of intellectual property trials concluded at the 278 prefecture-level cities level as a proxy indicator of the intensity of judicial protection of intellectual property in cities. First, we test the Pearson correlation coefficient between the IPDC dummy variable and the intensity of intellectual property protection in cities and find a significant positive correlation, with a correlation coefficient as high as 0.5492. Second, we calculate the intensity of intellectual property protection separately for model cities and non-demonstration cities. We find that the intensity of intellectual property protection in non-demonstration cities is 804.3704, while in model cities, it is as high as 3644.759. Therefore, the level of intellectual property protection in pilot demonstration cities is higher than that in non-demonstration cities.

### 4.2 Research sample and data sources

This study uses data from 2004 to 2019 for Chinese listed companies because Covid-19 may lead to data anomalies in 2020. In addition, we exclude the following samples. (i) companies lacking primary research data; (ii) companies in the financial sector; and (iii) ST, *ST, and PT companies. After processing, we obtain 22,759 firm-year observations, and the descriptive statistics of each variable are shown in Table 1. The data of listed companies used in this paper are obtained from the China Securities Market and Accounting Research (CSMAR) database and the China Research Data Service (CNRDS) database. the IPDC pilot list is obtained from the State Intellectual Property Office.

**Table 1:** Descriptive statistics.

Variables	Obs	Mean	Std. Dev.	Min	Max
GI	22759	3.223	32.618	0	1728
IPDC	22759	0.547	0.498	0	1
lnsize	22759	3.094	0.058	2.622	3.355
lnage	22759	1.997	0.916	0	3.434
property	22759	0.366	0.482	0	1
roa	22759	0.046	0.697	-6.776	108.366
lev	22759	0.414	0.334	-0.195	29.454
cash	22759	-0.171	11.906	-1785.882	24.187
audit	22759	0.057	0.231	0	1
herf	22759	34.855	14.99	0.29	89.99
er	22759	24.833	17.741	0	89.44
director	22759	10.092	2.629	4	26
indepen	22759	3.819	1.191	1	13

## 5. Empirical results

### 5.1 Difference-in-differences regression results

Table 2 reports the results of the baseline regression of IPDC on GI. Column (1) shows the results of the mixed OLS without control variables. Columns (2) and (3) are regression results controlling for year fixed effects and firm fixed effects, respectively. Column (4) is the result controlling for both fixed effects. Column (5) is the result after controlling for two-way fixed effects and all control variables. The baseline regression results indicate that the IPDC program has a positive incentive effect on the firm’s GI.

**Table 2:** Baseline regression results.

	(1)	(2)	(3)	(4)	(5)	(6)
	GI	GI	GI	GI	GI	GI
IPDC	0.744*	0.437	2.613***	1.165*	1.197**	1.201**
	(0.447)	(0.527)	(0.432)	(0.596)	(0.598)	(0.598)
lnsize					12.423	12.623
					(8.373)	(8.385)
lnage					-0.873	-0.885
					(0.596)	(0.597)
property					0.281	0.309
					(1.296)	(1.298)
roa					0.079	0.584
					(0.210)	(1.317)
lev					0.431	0.536
					(0.550)	(0.596)
cash					-0.001	-0.001
					(0.012)	(0.012)
audit					4.023**	4.017**
					(1.708)	(1.708)
herf					0.032	0.032
					(0.062)	(0.062)
er					-0.032	-0.031
					(0.047)	(0.047)
director					-0.171	-0.170
					(0.134)	(0.135)
indepen					0.293	0.292
					(0.245)	(0.245)
_cons	3.013***	3.181***	2.014***	2.804***	-34.123	-34.792
	(0.331)	(0.364)	(0.278)	(0.357)	(25.607)	(25.644)
Year effects	No	Yes	No	Yes	Yes	Yes
Firm effects	No	No	Yes	Yes	Yes	Yes
Observations	22743	22743	22619	22619	22619	22615
R-squared	0.001	0.001	0.626	0.626	0.626	0.627

Note:

***, **, * represent significant at the 1%, 5%, and 10% levels, respectively. Standard errors are in parentheses, the same below.

### 5.2 Robustness test results

#### 5.2.1 Parallel trend test and time trend analysis

**Fig 1:**
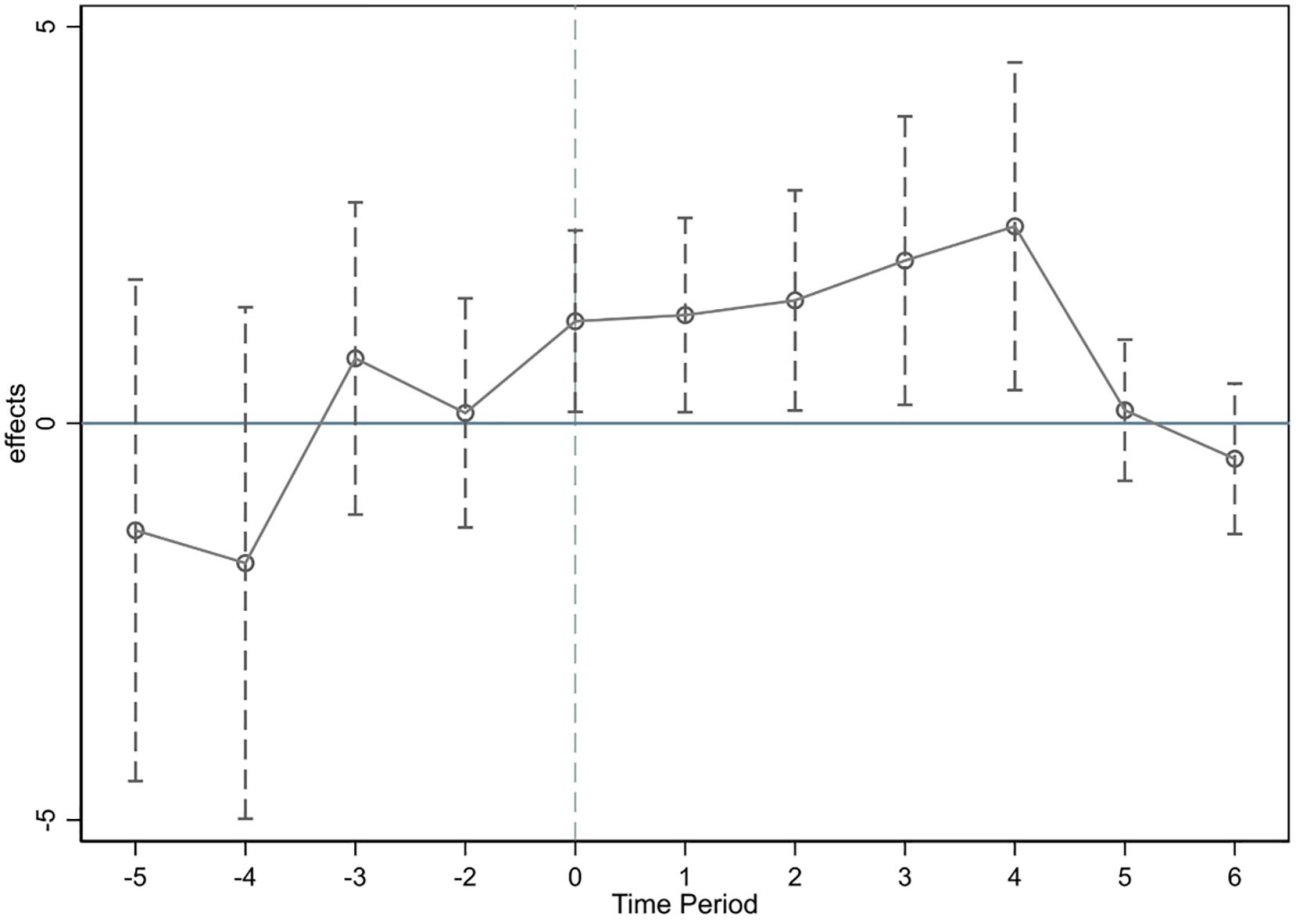
Parallel trend test.

DID estimation relies on the treatment and control groups to satisfy the parallel trend assumption. In the context of this study, this means that the trends in GI levels in the pilot and non-pilot cities prior to the pilot are consistent. Parallel trend tests and dynamic effects are shown in Fig 1. As can be seen from Fig 1, none of the coefficients were significant before IPDC implementation, i.e., the difference between the treatment and control groups was not significant before the IPDC project. The coefficients of the core explanatory variables are significantly positive in the period when IPDC is established, indicating that the IPDC program has a catalytic effect on the green innovation of enterprises after its implementation. In addition, it can also be seen from Fig 1 that the promotion effect of the IPDC program is long-term and the effect becomes larger as time advances until the fifth period afterward.

#### 5.2.2 PSM-DID model

**Fig 2:**
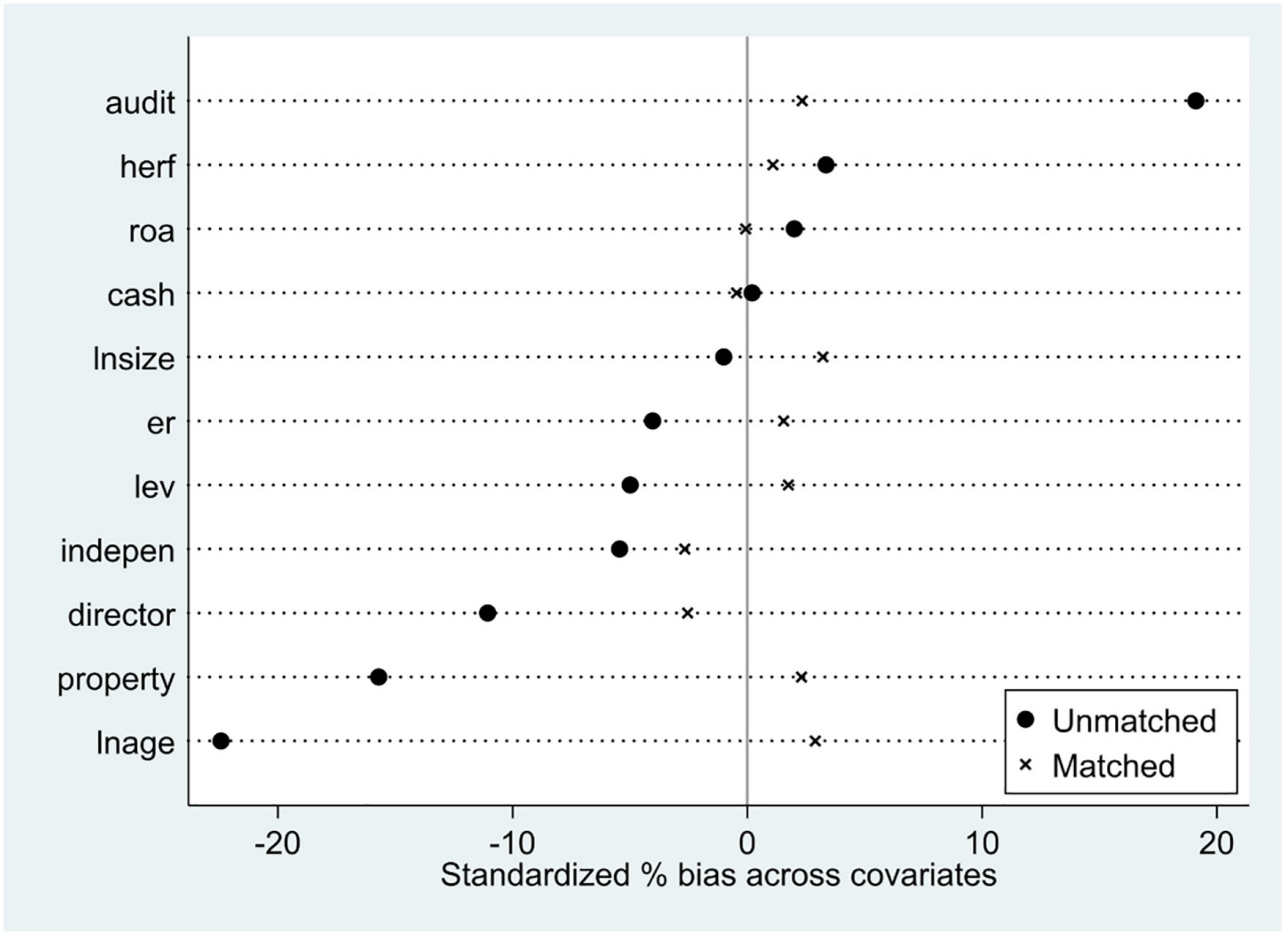
Normalized deviations before and after PSM.

Since the establishment of IPDC is subject to other factors, it is not strictly a natural experiment. This may cause selectivity bias problems and lead to biased estimates of the DID model. Therefore, this paper uses the multi-period propensity score matching method (PSM) to construct a period-by-period PSM, i.e., matching period-by-period on each cross-section of the panel data so that the experimental and control group firms are as insignificantly different as possible before the experiment. Specifically, this paper uses one-to-many nearest neighbor matching to find multiple individuals in the control group for each individual in the intervention group to match with. Meanwhile, the firm-level control variables are set as matching variables to re-estimate the effect of the IPDC program on firm green innovation. The normalized deviations before and after PSM are shown in Fig 2. It can be found that after PSM, the standard deviations of all control variables in both the treatment and control groups are reduced by a significant amount, indicating that the treatment and control groups are in a similar environment. The results in column (6) of Table 2 shows that the PSM-DID estimation is consistent with the DID estimation. After accounting for possible selectivity bias, the conclusions of this paper are not substantially changed, and IPDC does promote green innovation in firms.

#### 5.2.3 Contamination testing and robustness estimators

**Fig 3:**
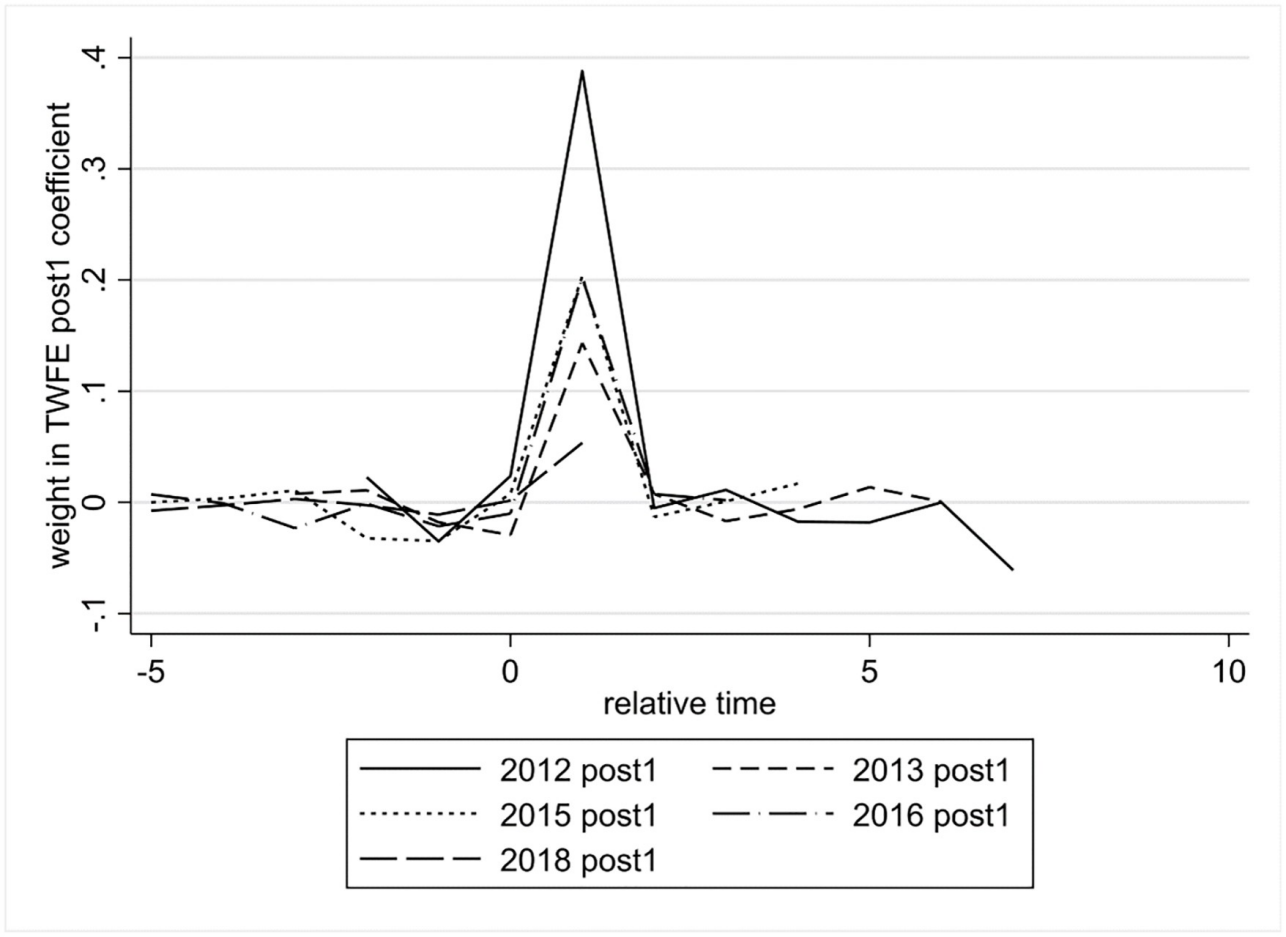
Weight decomposition of the TWFE estimator.

Recent econometric literature suggests that because multi-period DID vary in treatment time across units, the coefficients for a given lead or lag period may be contaminated by the effects of other periods [67–69]. Therefore, the use of two-way fixed effects (TWFE) estimators may produce bias or even yield opposite causal effects. To explore whether the green innovation effect of the IPDC program is contaminated by other periods, we decomposed the weights of the policy effect periods according to the method proposed by Sun and Abraham [67]. Fig 3 shows all the weights that make up the coefficients of the ex post first period. We find that all the weights except for the ex post one period are close to zero, which indicates that the policy effect of the ex post one period is little contaminated by the other periods.

To further confirm whether the TWFE estimator is biased, we reexamined the dynamic impact of IPDC on corporate green innovation using the alternative estimator without pollution proposed by Sun and Abraham [67], i.e., the IW estimator. Table 3 reports the dynamics of the TWFE and IW estimators. It can be found that the FE estimates and IW estimates are similar in magnitude and significance. This indicates that the results of the DID model in the baseline regression are robust, i.e., the IPDC program promotes corporate green innovation.

**Table 3:** Comparison of TWFE and IW estimated volumes.

	Pre5	Pre4	Pre3	Pre2	Current	Post1
TWFE	-1.348	-1.761	0.818	0.131	1.287*	1.364*
	(1.165)	(1.206)	(1.083)	(0.859)	(0.780)	(0.775)
IW	-0.907	-0.956	1.186	0.006	1.390*	1.198*
	(1.450)	(1.636)	(1.202)	(0.913)	(0.737)	(0.706)
	Post2	Post3	Post4	Post5	Post6	
TWFE	1.550**	2.051***	2.483***	0.166	-0.446	
	(0.758)	(0.728)	(0.782)	(0.883)	(0.847)	
IW	1.627*	2.092**	2.387**	-0.110	-0.484	
	(0.842)	(1.060)	(1.110)	(0.375)	(0.663)	

#### 5.2.4 Endogenous treatment

The adoption of quasi-natural experiments in this paper greatly mitigates the endogeneity of the model, but IPDC policies may not be entirely exogenous to shocks. Although PSM-DID has taken observable factors into account, there may still be unobservable factors affecting the approval of pilot cities. Therefore, this paper uses the instrumental variables approach to further reduce the endogeneity of the model. This paper uses the number of students enrolled in primary church elementary schools per 10,000 in each province in 1919 as an instrumental variable for the IPDC project. Fang and Zhao [70] finds that the Protestant ethic is highly correlated with the modern property rights system and can serve as a historical and cultural deposit that continues to influence the deep institutional environment in which the region’s market-based economic activity takes place. Therefore this historical data can be reflected in the local IPDC, satisfying the condition of relevance of the instrumental variable. Besides, the number of students in 1919 is less related to the innovation of modern enterprises, satisfying the exclusivity of the instrumental variable. The results of the instrumental variables method are shown in column (1) of Table 4, and it can be found that the conclusions still hold after addressing the issue of endogeneity.

**Table 4:** Robustness test.

	(1)	(2)	(3)	(4)	(5)	(6)
	GI	GI	GI	GI	GI	GI
IPDC	5.599**	1.228**	1.035*	1.197**	1.198**	1.197**
	(2.402)	(0.598)	(0.600)	(0.598)	(0.598)	(0.516)
Controls	Yes	Yes	Yes	Yes	Yes	Yes
Year effects	Yes	Yes	Yes	Yes	Yes	Yes
Firm effects	Yes	Yes	Yes	Yes	Yes	Yes
CET			2.058***			
			(0.697)			
FP				1.011		
				(1.001)		
LCC					0.609	
					(0.665)	
Observations	17734	22619	22619	22619	11084	22619
R-squared		0.627	0.627	0.627	0.627	0.627

#### 5.2.5 Winsorize data

To exclude estimation bias caused by outliers in the firm data, we shrink the continuous-valued variables at the firm level by the upper and lower 1% and re-regress the baseline model. As shown in column (2) of Table 4, the conclusion is still robust after winsorize.

#### 5.2.6 Excluding the interference of other pilot policies

To address the issue of climate change, China has also actively taken a variety of other measures before and after the IPDC pilot to promote green innovation in enterprises to reduce carbon emissions. First, since 2013, seven provinces and cities in Beijing, Tianjin, Shanghai, Chongqing, Guangdong, Hubei, and Shenzhen have launched carbon emissions trading pilots. Secondly, the National Development and Reform Commission has carried out three batches of pilot low-carbon provinces and regions and low-carbon cities from 2010 to 2017, aiming to promote low-carbon production and low-carbon consumption in cities. Finally, the Ministry of Finance decided to select some cities for financial subsidies during the 12th Five-Year Plan period to promote energy conservation and emission reduction in pilot cities by integrating financial resources and fiscal policies. The above pilot policies have been shown to be closely related to the green innovation behavior of enterprises. To remove the interference of these contemporaneous carbon emission reduction policies, this paper generates dummy variables for the occurrence or non-occurrence of carbon emissions trading (CET), energy efficiency fiscal policy (FP), and low carbon city (LCC) pilots, respectively, and adds them to the baseline regression model to further test the robustness of the results. The estimation results in columns (3)-(5) of Table 5 show that IPDC still has a significant contribution to firms’ green innovation even after considering other pilot policies.

**Table 5:** R&D spillover loss reduction mechanism.

	(1)	(2)
	High-Spillover	Low-Spillover
IPDC	2.869***	0.134
	(1.085)	(0.730)
Constants	-11.812	-59.169*
	(50.324)	(32.362)
Controls	Yes	Yes
Year effects	Yes	Yes
Firm effects	Yes	Yes
Observations	9133	13423
R-squared	0.688	0.540

#### 5.2.7 Robust standard error

This study further clusters the standard errors to the industry level and re-runs the regression, and as shown in column (6) of Table 4, the coefficients are still significant after using the robust standard errors. The above robustness tests indicate that the main findings of this paper are reliable and that the IPDC program significantly motivates the green innovation behavior of firms.

### 5.3 Mechanism test results

To test whether the IPDC program stimulates green innovation in firms by reducing the loss of R&D spillover, we construct an indicator to measure the extent of R&D spillover. Drawing on Raut [71], we use the Cobb-Douglas production function to measure the extent of R&D spillover across industries, as follows:

$${lnSale}_{i,t}=\alpha +\beta_{1}ln{R\&D}_{i,t}+\beta_{2}ln{R\&D}_{k,t}+\beta_{3}ln{Fixed}_{i,t}{+\beta }_{4}ln{Employee}_{i,t}+\varepsilon_{i,t}$$ (2)

Where *Sale*_*i*,*t*_ is the operating income in firm *i* in year *t*; *R&D*_*i*,*t*_ denotes the R&D stock in firm *i* in year *t*; *R&D*_*k*,*t*_ indicates the total R&D expenditure stock in year *t* for all companies in industry *k* except firm *i*; *Employee*_*i*,*t*_ is the number of employees of firm *i* in year *t*. The regression coefficient *β*_2_ indicates the extent to which the R&D investment of other firms in industry *k* contributes to the operating income of firm *i*. A large coefficient *β*_2_ represents a high degree of industry R&D spillover. Based on the data of all listed companies from 1990 to 2022, we conduct industry-specific regression, and finally get the regression coefficients of 85 industries to measure the R&D spillover intensity of the industries. To test whether the IPDC program has a greater impact on green innovation for firms in industries with more severe R&D spillover, we run group regressions according to the degree of R&D spillover in the industry to which the firm belongs. If the R&D spillover intensity of the industry to which the enterprise belongs is greater than the median of all industries, we define it as a high spillover group, and vice versa.

Table 5 demonstrates the impact of the IPDC program on green innovation for firms with different levels of R&D. It can be found that the IPDC program has a significant effect on green innovation only for firms with high R&D spillover effects. This indicates that firms concerned about R&D spillover losses from green innovation began to actively participate in green innovation after the establishment of IPDC. Therefore, hypothesis 1 is verified.

Similarly, we use group regressions to test whether the IPDC program promotes green innovation in firms by alleviating financing constraints. First, we use the KZ [72] and SA [73] indices, which are most frequently used in the existing literature, to measure the intensity of financing constraints. From columns (1)-(4) of Table 6, it can be found that the IPDC program is only effective in promoting green innovation for firms with high financing constraints. In addition, drawing on Hadlock and Pierce [73] and Wu and Tang [74], we also take the establishment years of firms as a robustness test for the financing constraint, since young firms have a high reliance on external financing. The results presented in columns (5) and (6) of Table 6 still support hypothesis 2.

**Table 6:** R&D spillover loss reduction mechanism.

	(1)	(2)	(3)	(4)	(5)	(6)
	High-KZ	Low-KZ	High-SA	Low-SA	High-Age	Low-Age
IPDC	2.309**	-0.185	4.561***	-0.255	1.075**	1.015
	(0.955)	(0.496)	(1.562)	(0.279)	(0.500)	(1.163)
Constants	-20.953	-55.504***	-37.994	-43.974***	-60.095**	-111.839**
	(55.195)	(19.231)	(83.413)	(13.721)	(30.054)	(53.274)
Controls	Yes	Yes	Yes	Yes	Yes	Yes
Year effects	Yes	Yes	Yes	Yes	Yes	Yes
Firm effects	Yes	Yes	Yes	Yes	Yes	Yes
Observations	13968	8035	8798	13429	11084	10113
R-squared	0.664	0.756	0.661	0.610	0.555	0.671

### 5.4 Heterogeneity test results

First, following the general literature, we measure the firm size in terms of the total assets of all firms and use their median as the basis for group regressions. Second, private and state ownership of firms are used to examine ownership heterogeneity. Finally, drawing on Liu, Lin [75]’s classification criteria, we examine the impact of life-cycle heterogeneity by dividing the firm’s life cycle into growth, maturity, and decline stages.

Table 7 reports the heterogeneous impact of the IPDC program on corporate green innovation under different firm characteristics. The results show that large-scale (LSEs), privately owned (POEs) and growth-stage enterprises (DSEs) are more likely to be stimulated by the IPDC program to carry out green innovation activities, which confirms hypothesis 3. These results imply that the IPDC program does not benefit all types of firms. Therefore, the government should formulate a targeted program to help these types of enterprises to carry out green innovation when promoting the IPDC program in the future.

**Table 7:** Heterogeneity test results.

	(1)	(2)	(3)	(4)	(5)	(6)	(7)
	LSEs	MSEs	SOEs	POEs	DSEs	MSEs	RSEs
IPDC	2.589**	0.011	1.468	0.968**	1.990**	-0.667	0.721
	(1.312)	(0.105)	(1.309)	(0.477)	(0.975)	(1.739)	(0.507)
Constants	-331.144***	-19.343***	-90.611	-41.960**	-84.727*	-65.835	-15.001
	(93.851)	(6.155)	(69.824)	(19.891)	(45.500)	(86.637)	(20.553)
Controls	Yes	Yes	Yes	Yes	Yes	Yes	Yes
Year effects	Yes	Yes	Yes	Yes	Yes	Yes	Yes
Firm effects	Yes	Yes	Yes	Yes	Yes	Yes	Yes
observations	10989	11334	8472	14061	9875	6398	4424
R-squared	0.627	0.593	0.640	0.567	0.682	0.661	0.683

## 6. Conclusions and policy implications

Based on data from Chinese listed firms from 2004–2019, this study examines for the first time the stimulating effect of the IPDC program on green innovation at the firm level. We develop theoretical hypotheses on the mechanism and the heterogeneous effects of the IPDC program on green innovation and conduct empirical tests based on the multi-period DID model. The results show that: First, China’s IPDC program significantly promotes corporate green innovation. This finding passes the parallel trend test and the conclusion still holds after excluding selective bias using the PSM-DID approach. Moreover, the DID estimators in this study are not contaminated and the robust IW estimators still support our results. Second, we theoretically analyze that an IPDC program would promote corporate green innovation by reducing R&D spillover losses and alleviating financing constraints. Our hypothesis is verified after constructing indicators of R&D spillover intensity and financing constraints. Third, we also find that the stimulus effect of the IPDC program is more pronounced in large, privately owned, growth-stage companies. However, small, state-owned, mature, and declining-stage firms will not be stimulated by the IPDC program to engage in more green innovation.

Based on these findings, we make the following policy recommendations. First, the protection of intellectual property rights is essential to stimulate green innovation in Chinese companies. The Chinese government should draw on the experience of the IPDC and continue to strengthen intellectual property protection nationwide. Second, the fear of loss of R&D spillovers and financing constraints are obstacles to green innovation by firms. Therefore, the government should intensify penalties for green innovation patent theft and plagiarism, and subsidize and reward companies with superior green innovation results. Third, the government should develop complementary policies for small, state-owned, declining firms to promote the stimulating effect of intellectual property protection on green innovation. For example, the government can protect small and medium-sized and declining-stage companies with additional subsidies to stimulate innovation. For state-owned enterprises, appropriate green innovation assessment with pressure can be conducted to stimulate vitality.

## Supporting information

S1 Data (DTA)
